# The determinants for death in hospital following moderate to severe traumatic brain injury in Australia

**DOI:** 10.1111/1742-6723.14562

**Published:** 2025-01-23

**Authors:** Gerard M O'Reilly, Afsana Afroz, Kate Curtis, Biswadev Mitra, Yesul Kim, Emma Solly, Courtney Ryder, Kate Hunter, Delia V Hendrie, Nick Rushworth, Jin Tee, Mark C Fitzgerald

**Affiliations:** ^1^ National Trauma Research Institute, Alfred Health Melbourne Victoria Australia; ^2^ Emergency and Trauma Centre, Alfred Health Melbourne Victoria Australia; ^3^ School of Public Health and Preventive Medicine, Monash University Melbourne Victoria Australia; ^4^ Susan Wakil School of Nursing and Midwifery, Faculty of Medicine and Health, University of Sydney Sydney New South Wales Australia; ^5^ Emergency Department Wollongong Hospital, Illawarra Shoalhaven Local Health District Wollongong New South Wales Australia; ^6^ The George Institute for Global Health, UNSW Sydney New South Wales Australia; ^7^ School of Translational Medicine, Monash University Melbourne Victoria Australia; ^8^ College of Medicine and Public Health, Flinders University Adelaide South Australia Australia; ^9^ School of Population Health, Curtin University Perth Western Australia Australia; ^10^ Brain Injury Australia Putney New South Wales Australia; ^11^ Neurosurgery, Alfred Health Melbourne Victoria Australia; ^12^ Trauma Service, Alfred Health Melbourne Victoria Australia

**Keywords:** injury, registries, trauma, traumatic brain injury

## Abstract

**Objectives:**

To establish the determinants of death in hospital for patients with moderate to severe traumatic brain injury (TBI) in Australia.

**Design, setting, participants:**

Retrospective analysis of Australia New Zealand Trauma Registry (ANZTR) data. Cases were included if they presented to a participating hospital between 1 July 2015 and 30 June 2020 and had an Abbreviated Injury Severity (AIS) score – head greater than 2.

**Main outcome measures:**

Death in hospital.

**Results:**

There were 16 350 patients. Their mean age was 51 years and 71% were male. After adjusting for measures of injury severity, there was an increased odds of in‐hospital death for patients whose injury occurred outside daylight hours or first mode of transport was road ambulance, who were not transferred from another hospital, had an endotracheal tube placed prior to definitive hospital arrival or received their definitive hospital care outside Victoria.

**Conclusion:**

Among people presenting to a major trauma hospital in Australia following moderate to severe TBI, there were multiple factors independently associated with death in hospital. The potentially modifiable determinants of in‐hospital death included out‐of‐hours access to emergency care, mode of transfer from the scene of the injury, prior facility care and pre‐definitive hospital endotracheal intubation.

## Graphical abstract

The effect of factors on death in hospital following moderate to severe traumatic brain injury (TBI) in Australia.

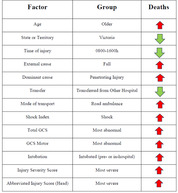

**Key findings**

- Factors increasing the odds of death in hospital following traumatic brain injury in Australia include being injured out‐of‐hours.
- Access to emergency care following traumatic brain injury needs to be addressed Australia‐wide.

## Introduction

Traumatic brain injury (TBI) is a major public health concern worldwide.^1^, ^2^ In Australia, TBI is a leading cause of injury‐related hospitalisations and deaths, imposing a considerable burden on individuals, families, and the healthcare system.^3^ TBI patients are typically classified as mild, moderate, or severe, with moderate to severe TBI (msTBI) associated with higher mortality rates and a worse long‐term prognosis.^4^, ^5^, ^6^

The determinants of death in hospital following msTBI remain complex and multifactorial. Age in particular is strongly associated with mortality and this association is important to understand given that older age groups now represent the majority of hospitalisations following msTBI in Australia.^7^, ^8^, ^9^ Injury severity measures are also consistently linked to patient outcomes, including the Glasgow Coma Scale (GCS) and Injury Severity Score (ISS).^6^, ^10^, ^11^, ^12^, ^13^

Understanding the factors that contribute to mortality in patients with msTBI is crucial for strengthening the health system and identifying opportunities for targeted interventions to improve patient outcomes. The Australian TBI National Data (ATBIND) Project was established to provide current, Australia‐wide data describing the incidence and determinants of outcomes from msTBI across Australia. Phase 1 of the ATBIND Project established the unchanging incidence of hospitalisation and death in hospital, following msTBI, across Australia.^9^ The aim of the present study (for Phase 2 of the ATBIND Project) was to identify, among people hospitalised following msTBI, the determinants of death in hospital.

## Methods

The detailed methods for the overall ATBIND Project protocol have been published previously.^14^ The following section describes the specific methods for the present study, a retrospective observational study of prospectively collected registry data.

Data from the Australia New Zealand Trauma Registry (ANZTR) were used for the present study.^15^ Cases are included in the ANZTR when the patient is assessed as having an ISS of more than 12 or died in hospital. The ISS is calculated by summing the squares of the Abbreviated Injury Scale (AIS) scores for the three most severely affected anatomical regions. The AIS is a ranking system for anatomical regions that ranges from 1 (Minor) to 6 (Maximum).^16^

Inclusion criteria for the present study were that ANZTR cases were admitted to one of the Australian ANZTR‐participating hospitals which provided data during the whole study period from July 1, 2015 to June 30, 2020 (Supporting Information Appendix S1), and had sustained a msTBI, defined as an AIS score greater than 2 for the head region,^17^ In general AIS scores of greater than 2 for the head region are assigned where there is at least serious injury to the head, including an injury to the brain parenchyma, cerebral blood vessels, base of skull fracture or major scalp loss or bleeding. Cases with incomplete data for the key variables of age, sex, and hospital mortality were excluded. The ANZTR has an ethically approved consent process which varies by Australian state or territory (e.g. opt out or waived).

## Statistical analyses

The primary outcome of the present study was death during the acute hospital stay (*vs* survival to hospital discharge). For the analysis described below, the candidate exposure variables were selected based on having existing data in the ANZTR, with overall completeness across the 5‐year study period of greater than 80%.

Symmetrical numerical data have been summarised using mean (standard deviation). Skewed numerical data or ordinal data have been summarised using median (interquartile range), and nominal data using frequencies (percentage). A univariable logistic regression analysis was conducted to determine the relationship between each of the candidate explanatory variables and the primary outcome variable (i.e. death in hospital). A multivariable logistic regression model was developed using stepwise variable selection to determine the independent predictors of the primary outcome (Supporting Information Appendix S2). Finally, the multivariable logistic regression analysis was repeated for two population subgroups: those who sustained an isolated msTBI and those with a non‐isolated msTBI (i.e. at least one moderately severe [or worse] injury elsewhere in the body). The magnitude of any association was presented as an odds ratio (OR) with 95% confidence intervals (95%CI). A *P*‐value of <0.05 was considered to be statistically significant. Data were prepared, analysed and graphed in STATA, version 17 (College Station, Texas, USA) statistical software.

## Ethics statement

The study received approval from The Alfred Hospital Research and Ethics Committee (project 670/21).

## Results

Over the duration of the study period (1st July 2015 to 30th June 2020), 16 350 cases met the inclusion criteria. Of these, there were 2437 (14.9%) in‐hospital deaths.

A data completeness check was conducted for all the variables and the variables with less than 20% missingness were selected for analysis. The results of the univariable analyses are provided in Table 1. Demographic and injury event variables which had a crude (unadjusted or univariable) association with death in hospital included: increasing age; injury location classified as remote or very remote Australia; fall; penetrating injury type; and place of injury recorded as residential institution. Being struck by a person or object as the external cause, and street or highway or workplace as the place of injury were protective against death in hospital in the univariable analysis.

**TABLE 1:** Results of univariable logistic regression analysis presenting association between demographic, injury event, injury severity and processes of care variables with in‐hospital death

Variable	Missing	Groups	All (*n* = 16 350)	Death (*n* = 2437)	Survival (*n* = 13 913)	Odds ratio (95% CI)	*P*‐value
	*n* (%)						
State or territory (*n* (%))	0 (0.0)	ACT	431 (2.6)	92 (21.4)	399 (78.6)	Reference	
		NSW	6316 (38.6)	960 (15.2)	5356 (84.8)	0.7 (0.5 to 0.8)	**0.001**
		NT	298 (1.8)	40 (13.4)	258 (86.6)	0.6 (0.4 to 0.8)	**0.007**
		QLD	1472 (9.0)	218 (14.8)	1254 (85.2)	0.6 (0.5 to 0.8)	**0.001**
		SA	1053 (6.4)	198 (18.8)	855 (81.2)	0.9 (0.6 to 1.1)	0.26
		VIC	4700 (28.8)	640 (13.6)	4060 (86.4)	0.6 (0.4 to 0.7)	**<0.001**
		WA	2.080 (12.8)	289 (13.9)	1791 (85.1)	0.6 (0.4 to 0.8)	**<0.001**
Remoteness of injury location (*n* (%))	1519 (9.3)	Major cities of Australia	10 689 (65.4)	1590 (14.9)	9099 (85.1)	Reference	
		Inner regional Australia	2973 (18.2)	437 (14.7)	2536 (85.3)	1.0 (0.9 to 1.1)	0.81
		Outer regional Australia	909 (5.6)	124 (13.6)	785 (86.4)	0.9 (0.7 to 1.1)	0.31
		Remote/very remote Australia	260 (1.6)	51 (19.6)	209 (80.4)	1.3 (1.0 to 1.9)	**0.04**
Age (years) (mean [SD])			(50.5 [26.1])	(60.4 [25.2])	(48.8 [25.8])	1.02 (1.01 to 1.02)	**<0.001**
Sex (*n* (%))	0 (0.0)	Male	11 644 (71.2)	1686 (14.5)	9958 (85.5)	Reference	
		Female	4706 (28.8)	751 (16.0)	3955 (84.0)	1.12 (1.02 to 1.23)	**0.01**
Time of injury (*n* (%))	0 (0.0)	00.00 to <08.00 hours	5229 (32.0)	763 (14.6)	4466 (85.4)	Reference	
		08.00 to <16.00 hours	5927 (36.2)	936 (15.8)	4991 (84.2)	1.1 (0.9 to 1.2)	0.08
		16.00 to <24.00 hours	5194 (31.8)	738 (14.2)	4456 (85.8)	0.9 (0.8 to 1.1)	0.58
External cause (*n* (%))	131 (0.8)	Transport	5706 (34.9)	822 (14.4)	4884 (85.6)	Reference	
		Fall	8069 (49.4)	1287 (16.0)	6782 (84.0)	1.1 (1.0 to 1.2)	**0.013**
		Struck by person or object	1623 (9.9)	145 (8.9)	1478 (91.3)	0.6 (0.5 to 0.7)	**<0.001**
		Other	821 (5.0)	138 (16.8)	683 (83.2)	1.2 (0.9 to 1.5)	0.07
Dominant cause (*n* (%))	20 (0.1)	Blunt	16 043 (98.1)	2353 (14.7)	13 690 (85.3)	Reference	
		Penetrating	260 (1.6)	58 (22.3)	202 (77.7)	1.7 (1.2 to 2.2)	**<0.001**
		Other	27 (0.2)	19 (70.4)	8 (29.6)	13.8 (6.0 to 31.6)	**<0.001**
Place of injury (*n* (%))	2957 (18.1)	Home	4690 (28.7)	811 (17.3)	3879 (82.7)	Reference	
		Residential institution	600 (3.7)	141 (23.5)	459 (76.5)	1.4 (1.2 to 1.8)	**<0.001**
		Street or highway	5021 (30.7)	716 (14.3)	4305 (85.7)	0.8 (0.7 to 0.9)	**<0.001**
		Workplace	1170 (7.2)	148 (12.6)	1022 (87.4)	0.7 (0.6 to 0.8)	**<0.001**
		Other	1.912 (11.7)	219 (11.5)	1693 (88.5)	0.6 (0.5 to 0.7)	**<0.001**
Mode of transport (*n* (%))	1388 (8.5)	Private or public vehicle	1433 (8.8)	49 (3.4)	1384 (96.6)	Reference	
		Air ambulance	2227 (13.6)	389 (17.5)	1838 (82.5)	5.9 (4.4 to 8.1)	**<0.001**
		Road ambulance	11 231 (68.7)	1833 (16.3)	9398 (83.7)	5.5 (4.1 to 7.3)	**<0.001**
		Other	71 (0.4)	19 (26.8)	52 (73.2)	10.3 (5.6 to 18.8)	**<0.001**
Transfer from other hospital (*n* (%))	27 (0.2)	No	10 804 (66.1)	1936 (17.9)	8868 (82.1)	Reference	
		Yes	5519 (33.8)	499 (9.0)	5020 (91.0)	0.5 (0.4 to 0.6)	**<0.001**
Pulse on arrival (/min) (mean [SD])	619 (3.7)		15 731 (87.9 [23.9])	2323 (90.9 [30.9])	13 408 (87.4 [22.4])	1.1 (1.0 to 1.2)	**<0.001**
SBP on arrival (mmHg) (mean [SD])	672 (4.1)		15 678 (134.2 [30.2])	2303 (130.9 (45.3)	13 375 (134.7 [26.7])	0.995 (0.994 to 0.997)	**<0.001**
Shock index (*n* (%))	822 (5.0)	Normal (≤1.0)	13 903 (85.0)	1770 (12.7)	12 133 (87.3)	Reference	
		Abnormal (>1.0)	1625 (10.0)	439 (27.0)	1186 (73.0)	2.5 (2.2 to 2.8)	**0.001**
GCS eye on arrival (*n* (%))	1386 (8.5)	Pain/verbal stimuli/spontaneous	10 803 (33.8)	659 (6.1)	10 144 (93.9)	Reference	
		No‐response/illegitimate	4161 (59.3)	1490 (35.8)	2671 (64.2)	8.5 (7.76 to 9.50)	**<0.001**
GCS voice on arrival (*n* (%))	1132 (6.9)	Confused/oriented/incomprehensible words/inappropriate words	10 784 (66.0)	629 (5.8)	10 155 (94.2)	Reference	
		No‐response/illegitimate	4434 (27.1)	1608 (36.3)	2826 (63.7)	9.2 (8.30 to 10.2)	**<0.001**
GCS motor on arrival (*n* (%))	1127 (6.9)	Withdraw to pain/localise pain/obeys command	11 118 (68.0)	731 (6.6)	10 387 (93.4)	Reference	
		No response/extension/flexion to pain/illegitimate	4105 (25.1)	1507 (36.7)	2598 (63.3)	8.2 (7.5 to 9.1)	**<0.001**
Total GCS on arrival (*n* (%))	1189 (7.3)	15	5922 (39.1)	149 (2.5)	5773 (97.5)	Reference	
		13–14	3840 (25.3)	256 (6.7)	3584 (93.3)	2.7 (2.3 to 3.4)	**<0.001**
		11–12	624 (4.1)	101 (16.2)	523 (83.8)	7.4 (5.7 to 9.7)	**<0.001**
		7–10	887 (5.8)	233 (26.3)	654 (73.7)	13.8 (11.1 to 17.2)	**<0.001**
		3‐6†	3888 (25.6)	1441 (37.1)	2447 (62.9)	22.8 (19.2 to 27.2)	**<0.001**
Time of intubation (*n* (%))	3016 (18.5)	No intubation	8161 (49.9)	488 (6.0)	7673 (94.0)	Reference	
		Intubated in hospital	4115 (25.2)	1292 (31.4)	2823 (68.6)	7.1 (6.4 to 8.1)	**<0.001**
		Intubated pre‐hospital	1058 (6.5)	312 (29.5)	746 (70.5)	6.6 (5.6 to 7.7)	**<0.001**
Disposition after ED (*n* (%))	755 (4.6)	Ward	7825 (47.9)	509 (6.5)	7316 (93.5)	Reference	
		ICU/HDU	5238 (32.0)	1015 (19.4)	4223 (80.6)	3.4 (3.1 to 3.9)	**<0.001**
		OR/OR to other destination	2314 (14.2)	540 (23.3)	1774 (76.7)	4.3 (3.8 to 4.9)	**<0.001**
		Other	218 (1.3)	9 (4.1)	209 (95.9)	0.6 (0.3 to 1.2)	0.16
Injury Severity Score (ISS) (*n* (%))	2 (0.0)	<13 (and death) – 24	9074 (55.5)	391 (16.0)	8683 (62.4)	Reference	
		25–40	6120 (37.4)	1559 (63.9)	4561 (32.8)	7.5 (6.7 to 8.5)	**<0.001**
		41–75	1154 (7.1)	487 (20.0)	667 (4.8)	16.2 (13.9 to 18.9)	**<0.001**
AIS head (*n* (%))	0 (0.0)	3	5454 (33.4)	284 (5.2)	5170 (94.8)	Reference	
		4	5996 (36.7)	457 (7.6)	5539 (92.4)	1.5 (1.3 to 1.8)	**<0.001**
		5	4877 (29.8)	1674 (34.3)	3203 (65.7)	9.5 (8.3 to 10.9)	**<0.001**
		6	23 (0.1)	22 (95.6)	1 (4.4)	400.4 (53.7 to 2981.7)	**<0.001**
Isolated *versus* non‐isolated TBI (*n* (%))	0 (0.0)	Isolated head	5964 (36.5)	1000 (41.1)	4964 (35.3)	Reference	
		Non‐isolated head	10 386 (63.5)	1437 (58.9)	8949 (64.7)	1.2 (1.1 to 1.4)	**<0.001**
Time between injury and hospital arrival (*n* (%))	99 (0.6)	0 to <1 h	2403 (14.8)	425 (17.7)	1978 (82.3)	Reference	
		1 to <24 h	12 055 (74.2)	1869 (15.5)	10 186 (84.5)	0.8 (0.7 to 0.9)	**0.01**
		≥24 h	1793 (11.0)	136 (7.6)	1657 (92.4)	0.4 (0.3 to 0.5)	**<0.001**
Time between hospital arrival and ED discharge (ED_LOS) (h) (median [IQR])	1644 (10.1)		4.2 (2.5, 7.5)	2.8 (1.4, 5.2)	4.5 (2.7, 7.8)		**<0.001** ^#^
Time between hospital arrival and ED discharge (ED_LOS) (h) (mean [SD])	1644 (10.1)		6.4 (17.2)	4.3 (8.2)	6.7 (18.3)	0.91 (0.89 to 0.92)	**<0.001**

*Note*: *P*‐values less than 0.05 are highlighted with bold font.

† Including ‘illegitimate’ GCS patients (i.e. intubated).

# *P*‐value derived using Wilcoxon Rank‐Sum test

Processes of care variables with a crude (unadjusted or univariable) association with death in hospital were: air and road ambulance as mode of transport from the scene of the injury; pre‐hospital and in‐hospital intubation; and ED disposition destination of intensive care unit (ICU) or operating theatre (OT). Conversely, increasing the time between injury and hospital arrival; interhospital transfer; and increasing ED length of stay were protective against subsequent death in the definitive hospital.

Injury severity variables with a crude (unadjusted or univariable) association with death in hospital were increasing heart rate; abnormal shock index; no response (or illegitimate) GCS eye, voice, or motor scores on arrival; total GCS on arrival less than 15; ISS of greater than 24; AIS head score of greater than 3; and non‐isolated TBI. Conversely, an increasing arrival SBP was protective against death in the definitive hospital.

The results of the multivariable logistic regression analysis, conducted to establish the independent (adjusted) associations with death in hospital, are presented in Table 2. The following demographic and injury event variables were found to have an independent association with in‐hospital death: increasing age; and the injury event being a fall or penetrating injury. However, the injury event occurring between 08.00 and 16.00 hours and the definitive care being delivered in Victoria were found to be protective against death in the hospital following msTBI.

**TABLE 2:** Results of multivariable logistic regression analysis for independent association with in‐hospital death

Variable	Groups	Odds ratio	95% CI	*P*‐value
Age (y)		1.05	1.04 to 1.05	<0.001
State or territory	ACT	Reference		
	NSW	–	–	–
	NT	–	–	–
	QLD	–	–	–
	SA	–	–	–
	VIC	0.8	0.7 to 0.9	0.009
	WA	–	–	–
Time of injury	00.00 to <08.00 hours	Reference		
	08.00 to <16.00 hours	0.8	0.7 to 0.9	0.034
	16.00 to <24.00 hours	–	–	–
External cause	Transport	Reference		
	Fall	1.5	1.2 to 1.9	<0.001
	Struck by person or object	–	–	–
	Other	2.3	1.5 to 3.5	<0.001
Dominant cause	Blunt	Reference		
	Penetrating	3.2	1.6 to 6.3	0.001
	Other	–	–	–
Place of injury	Home	Reference		
	Residential institution	–	–	–
	Street or highway	–	–	–
	Workplace	–	–	–
	Other	0.7	0.5 to 0.9	0.032
Mode of transport	Private or public vehicle	Reference		
	Air ambulance	–	–	–
	Road ambulance	1.4	1.1 to 1.8	0.002
	Other	8.2	2.6 to 25.8	0.001
Transfer from other hospital	No	Reference		
	Yes	0.5	0.4 to 0.6	<0.001
Shock index	Normal (≤1.0)	Reference		
	Abnormal (>1.0)	2.0	1.5 to 2.6	<0.001
Total GCS on arrival	15	Reference		
	13–14	1.8	1.3 to 2.5	<0.001
	11–12	3.8	2.4 to 5.9	<0.001
	7–10	5.6	3.8 to 8.3	<0.001
	3–6†	8.6	4.2 to 17.5	<0.001
GCS motor	Withdraw to pain/localise pain/obeys command	Reference		
	No response/extension/flexion to pain/illegitimate	1.9	1.0 to 3.6	0.049
Time of intubation	No intubation	Reference		
	Intubated in hospital	1.9	1.4 to 2.6	<0.001
	Intubated pre‐hospital	2.5	1.9 to 3.5	<0.001
Disposition after ED	Ward	Reference		
	ICU/HDU	–	–	–
	OR/OR to other destination	–	–	–
	Other	0.11	0.01 to 1.0	0.048
Injury Severity Score (ISS)	<13 (and death) to 24	Reference		
	25–40	–	–	–
	41–75	2.0	1.5 to 2.5	<0.001
AIS head	3	Reference		
	4	–	–	–
	5	5.4	4.5 to 6.4	<0.001
	6	–	–	–

† Including “illegitimate” GCS patients (i.e, intubated).

Additionally, several measures of care processes were independently associated with an increase in the odds of in‐hospital death. These included the use of road ambulance as the first mode of transport; and pre‐hospital or in‐hospital endotracheal intubation. Transfer from another hospital was protective against death in the definitive hospital.

Measures of injury severity which demonstrated an independent association with death in hospital included: response to painful stimulus worse than localisation (or illegitimate) for GCS‐Motor score on arrival; an abnormal shock index; a total GCS score of 14 or lower; an ISS of more than 40; and an AIS head score of 5.

The results of the multivariable logistic regression analyses to separately determine the independent factors associated with death in hospital for people presenting following an isolated msTBI and people with a non‐isolated msTBI, are summarised in Table 3. Although most of the independent associations with in‐hospital death identified in the overarching analyses were broadly consistent across both groups, there were several factors specific to just one or other of the subgroups (isolated *vs* non‐isolated msTBI). Specifically, for the isolated msTBI group, alone, there was an independent increase in the odds of death in hospital following penetrating injury mechanism and being injured in a remote area, while those who went directly from ED to the operating theatre had an independent reduction in the odds of death. Specifically, for the non‐isolated msTBI group, alone, there was an increase in the independent odds of death in hospital for those people who were injured in a residential institution, while among those who were injured in the higher population density states of Victoria and New South Wales and/or were transported by air ambulance, there was an independent reduction in the odds of death in hospital.

**TABLE 3:** Results of multivariable logistic regression analysis for independent association with in‐hospital death for people presenting with isolated *versus* non‐isolated msTBI

Variable	Groups	Isolated msTBI			Non‐isolated msTBI		
		Odds ratio	95% CI	*P*‐value	Odds ratio	95% CI	*P*‐value
Age (y)		1.06	1.05 to 1.07	<0.001	1.04	<1.04 to 1.05	<0.001
State or Territory	ACT	Reference			Reference		
	NSW	–	–	–	0.7	0.5 to 0.9	0.014
	NT	–	–	–	–	–	–
	QLD	–	–	–	–	–	–
	SA	–	–	–	–	–	–
	VIC	–	–	–	0.6	0.4 to 0.8	<0.001
	WA	–	–	–	–	–	–
External cause	Transport	Reference			Reference		
	Fall	2.8	1.6 to 4.9	<0.001	1.4	1.1 to 1.9	0.02
	Struck by person or object	2.5	1.2 to 5.3	0.02	–	–	–
	Other	3.6	1.6 to 8.4	0.002	2.3	1.3 to 4.0	0.002
Dominant cause	Blunt	Reference			Reference		
	Penetrating	3.9	1.4 to 11.1	0.01	–	–	–
	Other	–	–	–	–	–	–
Place of injury	Home	Reference			Reference		
	Residential Institution	–	–	–	3.2	1.8	<0.001
	Street or highway	–	–	–	–	–	–
	Workplace	–	–	–	–	–	–
	Other	–	–	–	0.7	0.4	0.04
Remoteness	Major Cities of Australia	Reference			Reference		
	Inner Regional Australia	–	–	–	–	–	
	Outer Regional Australia	–	–	–	–	–	–
	Remote/Very remote Australia	3.0	1.1 to 8.3	0.03	–	–	–
Mode of transport	Private or Public vehicle	Reference			Reference		
	Air Ambulance	–	–	–	0.7	0.5 to 0.9	0.02
	Road ambulance	–	–	–	–	–	–
	Other	–	–	–	8.3	1.4 to 47.8	0.02
Transfer from other hospital	No	Reference			Reference		
	Yes	0.5	0.3 to 0.6	<0.001	0.5	0.4 to 0.7	<0.001
Shock Index	Normal (≤1.0)	Reference			Reference		
	Abnormal (>1.0)	2.5	1.3 to 5.0	0.01	1.6	1.2 to 2.2	0.004
SBP (mmHg) on Arrival		–	–	–	1.0 (0.995)	1.0 to 1.0	0.03
Total GCS on Arrival	15	Reference			Reference		
	13–14	2.2	1.4 to 3.5	0.001	–	–	–
	11–12	7.0	3.6 to 13.5	<0.001	–	–	–
	7–10	7.7	4.4 to 13.4	<0.001	3.5	2.2 to 5.5	<0.001
	3–6†	28.2	15.7 to 50.8	<0.001	7.2	4.8 to 10.8	<0.001
Time of intubation	No intubation	Reference			Reference		
	Intubated in hospital	1.8	1.1 to 2.9	0.02	2.8	1.8 to 4.3	<0.001
	Intubated pre‐hospital	3.0	1.8 to 5.1	<0.001	2.9	1.9 to 4.5	<0.001
Disposition After ED	Ward	Reference			Reference		
	ICU/HDU	–	–	–	–	–	–
	OR/OR to other destination	0.6	0.4 to 0.9	0.009	–	–	–
	Other	–	–	–	–	–	–
Injury Severity Score (ISS)	<13 (and death) – 24	Reference			Reference		
	25–40	–	–	–	1.5	1.0 to 2.1	0.04
	41–75	–	–	–	2.5	1.7 to 3.9	<0.001
AIS head‡	3	Reference			Reference		
	4	0.1	0.0 to 0.2	<0.001	1.6	1.2 to 2.3	0.01
	5	0.5	0.2 to 1.0	0.04	6.6	4.6 to 9.5	<0.001
	6	–	–	–	–	–	–

† Including ‘illegitimate’ GCS patients (i.e. intubated).

‡ Note that if AIS head is 3 for a patient with an isolated TBI, then their ISS will be between 9 and 11; patients with an ISS <13 can only be included in the registry if they died during their definitive hospital stay. This explains why an AIS head of 4 or 5 is relatively protective compared to an AIS head of 3 (less odds of death) in this isolated TBI group.

## Discussion

The aim of the present study was to identify determinants of hospital mortality in patients with msTBI treated in major trauma centres across Australia. A number of demographic‐ and injury‐related factors were found to show an independent association with death in hospital. Older age was associated with higher odds of death, as were various characteristics of the injury event; msTBI sustained outside of working hours, and penetrating or falls‐related msTBIs, were associated with greater odds of death in hospital. Some processes of care measures demonstrated an independent association with death in hospital. After adjusting for measures of injury severity, patients whose injury occurred outside daylight hours, for whom the first mode of transport was road ambulance and the definitive hospital was outside Victoria, who were not transferred from another hospital or underwent endotracheal intubation prior to definitive hospital arrival had an increased odds of death in the definitive hospital. Unsurprisingly, various factors reflecting injury severity were also associated with greater odds of death, including GCS, GCS‐Motor, ISS, AIS head and SI.

There is a scarcity of Australia‐wide baseline data regarding outcomes from TBI, and determinants of outcomes. Identifying determinants is important both for the clinical management of individual patients and for improving patient care at a system level. Clinically, accurate prognostic predictions are important when communicating with patients and their families, as well as selecting treatment approaches. At a system level, these data are essential to identify modifiable factors to improve patient care, and to inform the development of strategies to ensure the most favourable patient outcomes.

Our results are in line with previous research on the demographic determinants of TBI outcomes. Increasing age has been consistently associated with both TBI mortality and longer‐term functioning.^7^, ^8^, ^18^, ^19^ The present study did not demonstrate that gender had an independent association with hospital mortality. Prior research on this topic is mixed, with the female gender reported as both a protective and a risk factor.^8^, ^20^ Regarding characteristics of the injury event, TBIs caused by falls were associated with significantly higher odds of death in hospital. This independent association of falls with mortality was maintained even when age was included in the model, indicating age does not completely account for this finding. Other health‐related factors not captured by these data such as frailty, which is associated with age but independently predicts mortality from injury, may both predispose individuals to falls, and contribute to poorer outcomes from TBI.^21^, ^22^, ^23^

Various indications of injury severity were associated with patient outcomes. More severe GCS, ISS, and AIS head scores predicted higher odds of death in patients with msTBI, as did shock on hospital arrival. The motor subscale of the GCS was the only GCS subscale independently associated with mortality, which is consistent with previous work.^24^ Importantly, each observed reduction in the arrival GCS (i.e. <15) was associated with higher odds of death.

Interestingly, even after adjusting for multiple anatomical (GCS‐total, GCS‐motor, ISS, AIS‐head) and physiological (SI) measures of injury severity, msTBI patients who were intubated prior to arrival at the definitive hospital were at an increased odds of death in hospital, as were those patients who were intubated at any time during their hospital stay. Whether the independent association of in‐hospital death with prior intubation is due to it being an additional measure of injury severity, even after adjusting for multiple other measures of injury severity, most measured at the time of definitive hospital arrival, needs further exploration.^15^ After adjusting for measures of injury severity, and whether or not the patient was transferred from another hospital, the time between injury and definitive hospital arrival was not associated with increased odds of death. This apparent contradiction to the adage that delays to definitive care increase the odds of death is likely explained by the study population or denominator being those who arrived at a major trauma facility, leading to a survivor bias.

It is important to consider which factors independently associated with in‐hospital mortality following msTBI may inform interventions and/or be modifiable. For example, one factor which could highlight modifiable aspects of TBI care is the timing of injury. MsTBI patients who sustained their injury during peak working hours had lower odds of death in hospital than patients injured at other times. Previous studies have also reported increased odds of death for trauma patients arriving at the hospital outside of standard working hours.^25^, ^26^ Further research would be needed to identify where access to quality out‐of‐hours patient care could be improved.

Differences in TBI hospital mortality were also identified between Australian states, with TBI patients in Victoria showing reduced odds of death relative to other states. The reason for this is unclear but may be in part because of geographical differences; as Victoria is a smaller state, there is a reduced distance to travel from any injury location to the nearest major trauma centre. This finding warrants further exploration.

Although in contrast to previous research,^21^, ^27^, ^28^ the present study did not demonstrate an independent association between death in hospital and remoteness of injury overall (noting that remoteness was an independent determinant of in‐hospital death for the *isolated* msTBI population), this may also be because of a survivor bias. That is, the ANZTR includes data from patients who survived to arrival at a major trauma centre. Some remote area patients with severe injuries will have died before reaching a trauma centre, and thus are not represented in these data. The consequence of this limitation is that we do not know how patients who do not reach a definitive hospital differ from the patients (included in the registry) who arrive at the definitive hospital alive. Paradoxically, if system‐wide access to the definitive hospital improves, the proportion of patients in the registry who die prior to discharge may increase. Furthermore, for those patients reaching the definitive hospital alive, the pre‐definitive hospital measures of physiology and processes of care suffer from suboptimal data completeness, limiting the capacity to test the importance of these variables.

Although the present study conducted some basic analyses for equity in terms of the primary outcome (e.g. including gender, age, geographic remoteness in the regression modelling), it was not able to analyse outcome disparities for Aboriginal and Torres Strait Islander people. For the five‐year study period examined, the registry had not yet commenced its system‐wide data collection for Aboriginal and Torres Strait Islander status.

The multivariable regression model used to identify the independent determinants of death in hospital is naturally limited in several ways. Only variables for which data are collected and relatively complete, can be included. Measures of statistical significance, while supported by consensus and determined pre‐hoc, are necessarily arbitrary. Where two or more variables overlap in terms of what they measure, the modelling process may remove the less informative variable(s) because of collinearity. The multivariable logistic regression model does not answer the question of which factors are modifiable (i.e. post‐injury care), and which are not, with the latter included as potential confounders to optimise the risk adjustment (i.e. injury severity as a risk for death). Furthermore, the analysis is not able to pinpoint the primary cause of death. For example, among the approximately two‐thirds of the study population with non‐isolated TBI, the TBI may not have been the primary cause or contributor to death in hospital.

## Conclusion

TBI is a major public health concern in Australia. In addition to various measures of increasing injury severity, the independent factors associated with an increased odds of death in hospital were: increasing age, falls, penetrating injuries, road ambulance from scene and intubation prior to hospital arrival. The independent factors associated with a decreased odds of death in hospital were: being injured during daylight hours, interhospital transfer, or receiving definitive care in a Victorian facility. All of these factors need to be scrutinised for being potentially modifiable and then targeted interventions (prevention, treatment and system‐wide) need to be identified, implemented and evaluated.

## Supporting information


**Appendix S1.** Australia New Zealand Trauma Registry (ANZTR) participating hospitals included in the study.

**Appendix S2.** Summary of statistical methods used to design the logistic regression model.

## Data Availability

The data that support the findings of the present study are held in a secure database (the ANZ Trauma Registry) according to appropriate data governance procedures and are only accessible through an appropriate ethics approval process.
