# Supplementary material for: The determinants for death in hospital following moderate to severe traumatic brain injury in Australia
Source: Emerg Med Australas. 2025 Jan 23;37(1):e14562. doi: 10.1111/1742-6723.14562 (PMC11755221; doi:10.1111/1742-6723.14562)
Supplement: Supplementary file 1 — Appendix S1. Australia New Zealand Trauma Registry (ANZTR) participating hospitals included in the study. [file EMM-37-0-s002.docx]

| **Australian Capital Territory**  Canberra Hospital  **New South Wales**  Children’s Hospital Westmead  John Hunter Children’s Hospital  John Hunter Hospital  Liverpool Hospital  Royal North Shore Hospital  Royal Prince Alfred Hospital  St George Hospital  St Vincent’s Hospital  Sydney Children’s Hospital  Westmead Hospital  **Northern Territory**  Royal Darwin Hospital  **Queensland**  Gold Coast University Hospital  Queensland Children’s Hospital  Royal Brisbane and Women’s Hospital  **South Australia**  Flinders’ Medical Centre  Royal Adelaide Hospital  Women’s and Children’s Hospital  **Victoria**  The Alfred  Royal Children’s Hospital  Royal Melbourne Hospital  **Western Australia**  Perth Children’s Hospital  Royal Perth Hospital |
| --- |

**Appendix 1:** Australia New Zealand Trauma Registry (ATR) participating hospitals included in the study.
