# Supplementary material for: The determinants for death in hospital following moderate to severe traumatic brain injury in Australia
Source: Emerg Med Australas. 2025 Jan 23;37(1):e14562. doi: 10.1111/1742-6723.14562 (PMC11755221; doi:10.1111/1742-6723.14562)
Supplement: Supplementary file 2 — Appendix S2. Summary of statistical methods used to design the logistic regression model. [file EMM-37-0-s001.docx]

**Appendix 2:** Summary of statistical methods used to design the logistic regression model

The dependent variable for the regression modelling was the primary outcome for the study. This was defined a death during the acute hospital stay (versus survival to discharge).

The candidate exposure variables for testing an association (as modifiable factors and/or potential confounders) were selected according to the following criteria:

1. Collected in the Australia New Zealand Trauma Registry
2. A completeness proportion of greater than 80% over the 5-year study period

All variables meeting the above criteria were tested, in turn, for a crude (unadjusted) association with the dependent variable (i.e. death during the acute hospital stay), using univariable logistic regression analyses. The results, for all the candidate exposure / independent variables have all been reported in Table 1 with the measure of association being the Odds Ratio, with the 95% Confidence Interval and P-Value for that Odds Ratio as the measures of statistical significance.

To determine the independent (adjusted) association between the dependent variable (the primary outcome of death during the acute definitive hospital stay) and all of the candidate exposure variables considered in the univariable analyses, a multivariable logistic regression model was developed.

For the purposes of this analysis, as all the variables could be defined as have a plausible association with the dependent variable (death in hospital) as a modifiable factor and/or a potential confounder, all of these variables were included in the initial “full” model. The stepwise multivariable logistic regression modelling procedure used progressively (i.e. in a stepwise fashion) removes variables from the model in sequence according to how far removed their measure P-value is from the chose measure (cutoff) of statistical significance, which in this case was chosen as a P-value of 0.05. The final multivariable regression model contains all of the remaining candidate exposure variables (modifiable factors and / or potential confounders) for which the independent (adjusted) association with the dependent variable (death in hospital) is deemed statistically significant (i.e. real) according to the pre-hoc (pre-defined) definition of statistical significance (P-value less than 0.05). The results for the final multivariable regression model have been provided in Table 2.
